# Cost-utility analysis of palivizumab in Italy: results from a simulation model in the prophylaxis of respiratory syncytial virus infection (RSV) among high-risk preterm infants

**DOI:** 10.1186/1824-7288-35-4

**Published:** 2009-02-25

**Authors:** Gaetano Chirico, Roberto Ravasio, Urbano Sbarigia

**Affiliations:** 1 Neonatologia e Terapia Intensiva Neonatale, Spedali Civili, Brescia, Italy; 2 Wolters Kluwer Health/Adis International Ltd, Milan, Italy; 3 Health Economics – Market Access & Health Policies, Abbott, Italy

## Abstract

**Introduction:**

The aim of this study was to assess the cost-utility of palivizumab versus no prophylaxis in the prevention of respiratory syncytial virus infection among high-risk preterm infants.

**Methods:**

We used and adapted a pre-existent model in which two cohorts of patients received palivizumab or no prophylaxis. The patients were followed for their expected lifetimes. The economic evaluation was conducted from the perspective of the Italian National Health Service. We considered Life-Years Gained (LYGs), Quality-Adjusted Life-Years (QALYs) and direct medical costs (pharmacological treatment, hospitalization, recurrences for wheezing, etc.). LYGs and QALYs were based on the results of a double blind cohort study with prospective follow-up and direct medical costs were based on Italian treatment patterns. Benefits and costs were discounted at 3%. Costs were assessed in 2007 Euros. Sensitivity and threshold analysis on key clinical and economic parameters were performed.

**Result:**

For the two cohorts, the expected life-years (per patient) with palivizumab versus no prophylaxis were 29.842 and 29.754 years, respectively. Quality-adjusted life years (per patient) with palivizumab were 29.202, and for no prophylaxis were 29.043. The expected cost (per patient) was € 6,244.20 with palivizumab and € 4,867.70 with no prophylaxis. We calculated for palivizumab versus no prophylaxis the incremental cost per LYG and per QALY gained. It was € 15,568.65 and € 8,676.74, respectively.

**Conclusion:**

This study suggests that, compared with no prophylaxis, palivizumab is cost-effective in the prevention of respiratory syncytial virus infection among high risk preterm infants.

## Background

Respiratory Syncytial Virus (RSV) is the most common cause of viral respiratory tract infections in infancy. [1] The common presentation of RSV on infants are lower respiratory tract infections such as pneumonias and bronchiolitis occurring usually during the first two years of life. [2-6]

In Italy about 4–5,000 RSV-infected, high-risk, preterm infants (gestational age < 36 weeks, with or without bronchopulmonary dysplasia [BPD]) are hospitalized every year. A proportion of these infants require admission to intensive care units due to severity of the condition and the level of care needed. [7] Mortality rates in hospitalized infants are high, reaching almost 4% during the first year of life. [8]

Several prospective clinical studies have moreover demonstrated a strong correlation between RSV and recurrent wheezing/asthma episodes. These studies have more specifically noted that the airway hyperreactivity rate is higher by 50%–100% in RSV-infected infants compared to non-infected children.[9] Presence of recurrent wheezing episodes was observed through the age of 11 years, possibly extending throughout early adolescence.[4,6]

In these past few years, the launch of the intramuscular humanized monoclonal antibody, palivizumab, has added to the therapeutic options available for RSV prophylaxis. This molecule demonstrated clinical efficacy and satisfactory tolerability. [10-13]

In consideration of the above, Simoes et al. [14] conducted a double blind cohort study with prospective follow-up to verify whether administration of palivizumab in high-risk preterm infants was capable not only of preventing RSV infections but also of reducing the number of possible subsequent recurrent wheezing episodes. Incidence rates of physician-diagnosed recurrent wheezing episodes over the 2-year duration of the study in preterm patients not requiring RSV hospitalization were noted to be significantly lower in the group of preterm infants receiving palivizumab prophylaxis rather than in children with no prophylaxis, showing rates of 8% *versus *16%, respectively (p = 0,011).

In consideration of the findings obtained by Simoes et al. [14], it was considered appropriate to update the results of a prior cost-effectiveness analysis [15] comparing palivizumab versus absence of prophylaxis in the prevention of RSV infections in preterm infants of different gestational ages (less than 33 weeks, and 33 to 35 weeks) with or without complications (BPD).

The present study intended to assess quality-adjusted and non-adjusted incremental cost-effectiveness ratios per year of life for palivizumab versus non-prophylaxis in the prevention of RSV infections in high-risk preterm infants.

## Methods

### Background

The incremental cost-effectiveness analysis (Incremental Cost Effectiveness Ratio – ICER) was conducted from the perspective of the National Health System (NHS) taking into account the direct health care expenditure assessed in 2007 euros. Life-years (LY) and QALYs (*Quality-Adjusted Life-Years*) are the two outcomes assessed by the decision-making model. As a lifetime horizon was used in the study, a 3% discount rate was applied both to expenditure and outcomes. In particular, costs associated with prophylaxis and absence of prophylaxis were assessed for the first two years after enrollment.

### Model

**Figure 1:**
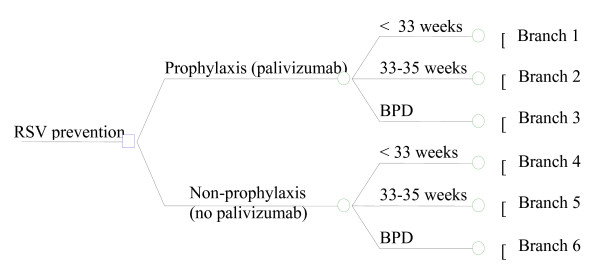
**General structure of the decision-making tree model**.

In order to estimate cost-effectiveness ratios for palivizumab versus non-prophylaxis, a pre-existing decision-making tree model [15] (Figure 1) was updated based on the results published by Simoes et al. [14]

**Figure 2:**
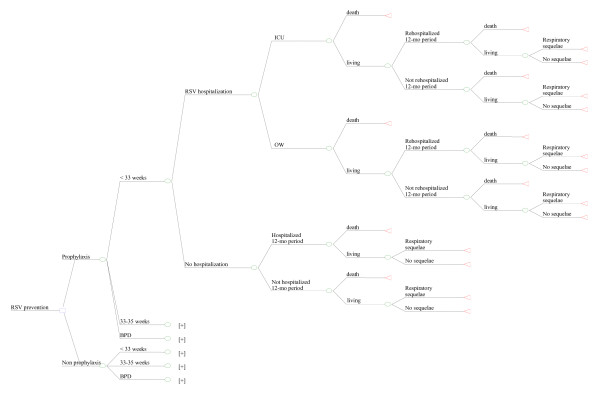
**Detail of the "Prophylaxis, < 33 weeks" branch**.

Figure 2 describes the development of only one of the possible branches foreseen by the decision-making model ("Prophylaxis – < 33 weeks"), since branches are all equivalent in terms of structure and are differentiated only in terms of the different odds assigned to the probabilistic nodes and the different health care expenditure.

The course of the "Prophylaxis, < 33 weeks" branch foresees that infants may or may not develop an RSV infection with ensuing hospitalization within 12 months post administration of prophylaxis. Based on the severity of the pediatric patient's conditions, he/she may be hospitalized in the ordinary ward (OW) or in the Intensive Care Unit (ICU). In either case, OW or ICU, preterm infants might die due to RSV. Subsequently, after the first hospitalization and once again within the 12 months following administration of prophylaxis, survivors might experience infection-related sequelae requiring rehospitalization or leading to death. Finally, patients who survive are followed for further 12 months to assess chances of developing recurrent wheezing episode 24 months after prophylaxis with palivizumab.

On the other hand, in the event pediatric patients are not immediately hospitalized due to RSV infection, the model foresees the possibility of hospitalizations (and the relating respiratory infection-related mortality rate) within the 12-month period following enrollment (administration of prophylaxis) and of recurrent wheezing episodes in the subsequent 24 months.

### Probability of events

The probabilities of occurrence of events considered in the present study (Tables 1 and 2) are equal to those used in the original model [15], except for the probability of developing recurrent wheezing in the 24 months of follow-up after the enrolment.

**Table 1:** Risk class and probability of hospitalization

	**Prophylaxis**			**Non prophylaxis**		
	
**Probability of occurrence**	***33–35 wks***	***< 33 wks***	***BPD***	***33–35 wks***	***< 33 wks***	***BPD***
**Risk class**						

- patient rate	11.0%	70.0%	19.0%	11.0%	70.0%	19.0%

**Probability of hospitalization**						

- ordinary ward	1.5%	2.0%	5.6%	9.8%	10.3%	18.4%

- Intensive therapy unit	1.3%	1.3%	1.3%	3.0%	3.0%	3.0%

From: Ravasio R et al. [^15^]

**Table 2:** Probability of events following the first hospitalization and number of events

	**RSV-hospitalized subjects**			**Subjects without RSV-hospitalization**		
	
**Probability of occurrence**	***33–35 wks***	***< 33 wks***	***BPD***	***33–35 wks***	***< 33 wks***	***BPD***
**Probability of events following the first hospitalization**						

- hospitalization during the first 12 months after enrolment	100.0%	100.0%	100.0%	60.0%	60.0%	60.0%

- mortality throughout the first 12 months	4.0%	4.0%	4.0%	0.8%	0.8%	0.8%

- probability of developing recurrent wheezing throughout the 24 months following enrolment with palivizumab [^14^]	17.0%*	17.0%*	17.0%*	8.0%	8.0%	8.0%

- probability of developing recurrent wheezing throughout the 24 months following enrolment without palivizumab [^14^]	17.0%	17.0%	17.0%	16.0%	16.0%	16.0%

**Events**						

- no. of hospitalizations due to respiratory causes during the first 12 months after enrollment	2.89	5.40	5.40	1.28	1.00	1.00

From: Ravasio R et al. [^15^]; *Conservative assumption based on study data published

The latter clinical finding was calculated based on the results published by Simoes et al. [14]. The probability of developing recurrent wheezing in patients who were not hospitalized for RSV was noted to be 8% for palivizumab-treated subjects and 16% in subjects who did not receive prophylaxis. The data published by Simoes et al. [14] made it possible to calculate a 17% probability of developing recurrent wheezing in subjects who where hospitalized for RSV and who did not receive prophylaxis (13/76 patients). In order to complete the population of the present simulation model, the decision was taken to set at a conservative 17% the odds of developing recurrent wheezing in the 24 months of follow-up after the enrolment also for RSV-hospitalized patients treated with palivizumab; the odds being equal to the probability for patients without prophylaxis (Table 2).

### Use of Resources

As with the original model, the use of resources refers to administration of prophylaxis, any hospitalizations required to treat RSV infections and treatment for recurrent wheezing.

### Outcome

The present decision-making model, in accordance with the original study [15], has provided an estimate of life-years based on the various mortality odds associated with the different probabilistic nodes for patients with or without prophylaxis.

Even quality-adjusted life-years (QALY) were estimated based on the method used in the original study. [15] They were calculated by adding, for the expected average life span, quality-weighted duration of the disease associated with the presence or absence of RSV [16,17](Table 3).

**Table 3:** Quality of life

**Variable**	**Value**
Health-related QoL score* (range 0–1)	
No RSV hospitalization	0.950
RSV hospitalization	0.880

From: Greenough A et al. [16], calculations based on Health Utility Index 2: Torrance GW et al. [17].

*QoL = Quality of Life

### Costs

The € 3,099.84 cost for prophylaxis with palivizumab was calculated based on NHS reimbursements rates [18], and on the administration scheme foreseeing a recommended dosage of 15 mg per kilogram. The model is based on a palivizumab administration scheme of one 50 mg vial/month for the first three months and one 100 mg vial/month for the subsequent two months, for an overall cost of € 3,099.84. The overall dosage was calculated based on a conservative usage hypothesis, in other words expenditure includes also any unused active principle.

Appreciation of hospitalization-related costs was based on the Interregional tariff agreement (*Tariffa Unica Convenzionale*). [19] In particular, with respect to RSV infection hospitalization in ordinary wards, DRG 98 was taken into account – Bronchitis and asthma, age < 18 years – equal to € 1,328.22, whereas for RSV admission to Intensive Care, reference was made to DRG 475- Diagnoses relating to the respiratory system with mechanical ventilation – equal to € 8,158.90. Finally for hospitalizations occurring for lower respiratory tract infections subsequent to the first hospitalization, DRG 81 – Respiratory infections and inflammations, age < 18 year – equal to € 3,729.54. [15]

Since no data are available in published studies, the annual mean cost for the treatment of a pediatric patient with recurrent wheezing was appreciated based on the results of the SIRIO study [20], which had calculated the annual mean cost of an adult patient suffering from recurrent wheezing with respect to the Italian clinical setting (Table 4).

**Table 4:** Annual mean cost for a patient with asthma

**Expenditure Breakdown**	**Mean cost per Patient (€)**
Pharmacological therapy	457.82

Hospitalizations (ordinary admission and Day Hospital)	461.87

Admissions to Emergency Room	5.11

Visits	112.26

Examinations	127.76

Specific immune therapy	31.86

Other	30.21

**Annual mean cost**	**1,226.88**

### Sensitivity analyses

In order to test the robustness of the estimates resulting from the decision-making model [21] a series of univariate analyses were performed on the variables producing the greatest impact on the results of the simulation model: palivizumab dosage scheme, annual mean cost for a patient with recurrent wheezing and probability of developing recurrent wheezing within 24 months post-enrollment. In the first case (palivizumab dosage) the univariate analysis was conducted based on the assumption of a prophylaxis scheme with five monthly 100 mg injections, contrary to the base case scenario (three 50 mg administrations and two 100 mg administrations). The annual mean cost for a patient with recurrent wheezing changed based on the limits of the respective Confidence Interval (CI 95%, € 1,054.34 – € 1,399.42), whereas the probability of developing recurrent wheezing within 24 months post-enrolment was adjusted by ± 10% compared to its base value [21] assuming the worst case scenario for palivizumab (+10% [least advantageous] compared to the base value for the "Prophylaxis" branch and -10% [more advantageous] for the "Non prophylaxis" branch).

Finally, a threshold analysis was conducted on the probability of an RSV hospitalization occurring in patients receiving palivizumab prophylaxis. This analysis intended to identify which value of this parameter would show an incremental cost per life year (quality weighted and non-quality weighted) equal to € 50.000 in the simulation model, considering the latter value to represent the threshold below which a therapy would be considered acceptable. [22]

## Results

### Life years (LY) and QALY (3% discount)

Patients receiving palivizumab prophylaxis show better efficacy results measured in terms both of LYs (+ 0.088) and of QALYs (+ 0.159) [Table 5]. Such an advantage, versus the absence of prophylaxis comparator, is maintained even when analyzing the results for the comparisons on patient subgroups (BPD, < 33 weeks, 33–35 weeks) [Table 5].

**Table 5:** Results: LY and QALY (3% discount)

**Parameter**	**Prophylaxis**	**Non prophylaxis**	**Difference**
**LY**			

**Total**	**29.842**	**29.754**	**0.088**

*- BPD*	29.813	29.694	0.119

*- < 33 weeks*	29.849	29.771	0.078

*- 33–35 weeks*	29.854	29.776	0.078

***QALY***			

**Total**	**29.202**	**29.043**	**0.159**

*- BPD*	29.173	28.985	0.188

*- < 33 weeks*.	29.209	29.060	0.150

*- 33–35 weeks*	29.214	29.065	0.149

### Total treatment costs (3% discount)

The annual mean cost per patient receiving prophylaxis (€ 6,244.20) exceeds by € 1,376.50 the cost estimated for a non-prophylaxis patient (€ 4,867.70) [Table 6]. The same result is apparent also in comparisons across patient subgroups, with differences between the two therapeutic options ranging from € 513.47 to € 2,220.09.

**Table 6:** Results: mean cost per patient (3% discount)

	**Mean cost per treated patient**		
	
**Parameter**	**Prophylaxis**	**Non prophylaxis**	**Difference**
*- BPD*	6,517.26	6,003.79	513.47

*- < 33 weeks*	5,819.65	4,429.13	1,390.52

*- 33–35 weeks*	6,199.79	3,979.70	2,220.09

**Total**	**6,244.20**	**4,867.70**	**1,376.50**

### Incremental cost-effectiveness ratio

In terms of overall data ("total mean"), the incremental cost effectiveness ratio per LY estimated by the model was equal to € 15,568.65, and that per QALY was € 8,676.74 (Table 7).

**Table 7:** Results: ICER (3% discounted costs and outcomes)

**Parameter**	**ICER**
**LY**	

**Total (mean)**	**15,568.65**

*- BPD*	4,332.29

*- < 33 weeks*	17,885.86

*- 33–35 weeks*	28,417.08

***QALY***	

**Total (mean)**	**8,676.74**

*- BPD*	2,731.81

*- < 33 weeks*	9,380.00

*- 33–35 weeks*	14,937.32

With respect to the secondary analysis conducted for the three subgroups, ICERs were observed to range between € 4,332.29 – € 28,417.08 per LY, and from € 2,731.81 to € 14,937.32 for QALYs.

### Sensitivity analysis

#### Univariate analysis

Table 8 illustrates the results of the sensitivity analysis conducted by adjusting the base values as per the palivizumab dosage scheme, the annual mean cost of a patient with recurrent wheezing patient and the probabilities of developing recurrent wheezing within 24 months post-enrolment. The incremental cost effectiveness ratios per LY or per QALY estimated by all the univariate analyses show values lower than the internationally accepted € 50,000 threshold [22].

**Table 8:** Univariate sensitivity analysis

**Parameters**	**ICER per LY (€)**	**ICER per QALY (€)**
**Palivizumab dosage scheme**		
Total	25,352.07	14,129.26
BPD	12,532.56	7,902.66
< 33 weeks	30,387.26	15,936.20
33–35 weeks	40,857.49	21,476.58
**Annual mean cost per patient with recurrent wheezing**		
Total	15,437.90 – 15,699.62	8,603.88 – 8,749.74
BPD	4,225.56 – 4,438.94	2,664.51 – 2,799.06
< 33 weeks	17,717.48 – 18,054.36	9,291.70 – 9,468.37
33–35 weeks	28,248.76 – 28,585.65	14,848.85 – 15,025.93
**Probability of developing recurrent wheezing within 24 months from enrollment**		
Total	15,857.51	8,837.74
BPD	4,577.05	2,886.15
< 33 weeks	18,253.99	9,573.06
33–35 weeks	28,782.64	15,129.48

Amongst those taken into account, the parameter that most affects the variability of incremental cost-effectiveness ratios is the palivizumab dosage scheme. When mean data ("Total") are referred to, the univariate analyses conducted on this parameter show an increase of ICER versus base values by 62.8% both per LY and QALY (Table 8).

#### Threshold analyses

**Figure 3:**
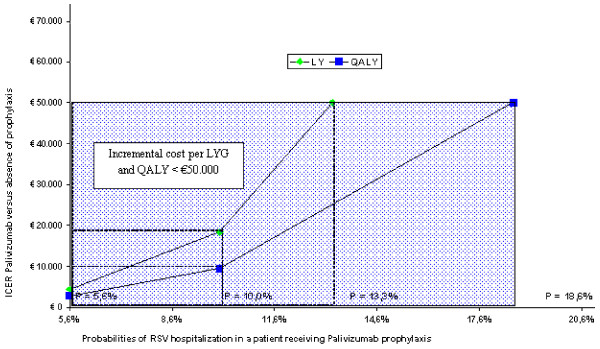
**Threshold analysis: group of preterm BPD infants**.

**Figure 4:**
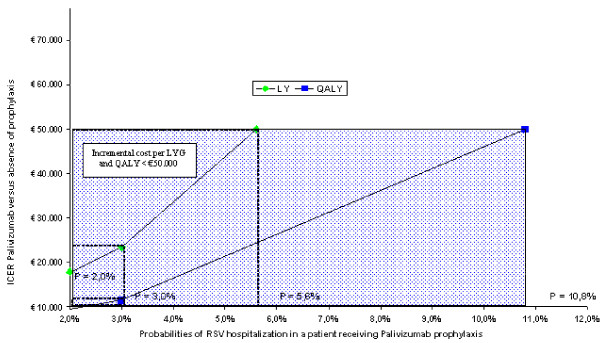
**Threshold analysis: group of infants with gestational < 33 weeks**.

**Figure 5:**
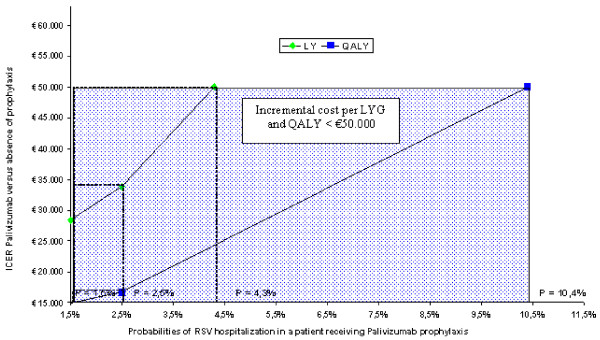
**Threshold analysis: group of infants with gestational age between 33 and 35 weeks**.

Figures 3, 4 and 5 illustrate the results of the threshold analyses. The three analyses were based on a simulation reducing efficacy of palivizumab, therefore increasing the probabilities of an RSV hospitalization in prophylaxis-treated patients, differentiating between preterm infants with BPD, gestational age < 33 weeks and gestational age between 33 and 35 weeks.

In the first case (preterm BPD infants) (figure 3) the threshold level (for hospitalizations) at which palivizumab shows an incremental cost per LY of € 50,000 is 13.3% (increased by 137.5% compared to the 5.6% base value), whereas for QALYs such value rises to 18.6% (+ 232.1% compared to the base value).

In the second case (infants with gestational age < 33 weeks) (figure 4) the threshold level at which palivizumab shows incremental values per LY of € 50,000 is 5.6% (increased by 180% compared to the 2% base value), rising to 10.8% for QALYs (+440% compared to base values).

Finally, the third case (children whose gestational age ranges between 33 and 35 weeks) (figure 5) points out a 4.3% threshold level at which Palivizumab shows an incremental cost per LY of € 50,000 (increased by 186.7% compared to the 1.5% base value), whereas said rate rises to 10.4% for QALYs (+ 593.3% compared to base value).

## Discussion

The present analysis assessed efficacy and costs of prophylaxis with palivizumab versus absence of prophylaxis in the prevention of RSV infections in high-risk preterm infants. The (incremental) cost-effectiveness analysis was conducted from the perspective of the National Health System, updating and adapting a pre-existing lifetime decision-making model [15]. We wish to point out that as in the previously published model, RSV infection mortality during the first hospitalization was estimated based on the results of the IMPACT study: mortality rates were noted to be 0.2% and 0.0% in subjects who received palivizumab prophylaxis and subjects who did not receive prophylaxis, respectively [15]. Other probability rates of occurrence of the events investigated in the present study are also the same ones used in the original model [15], with the exception of the odds of developing recurrent wheezing within 24 months post-enrolment, data deriving from Simoes study [14].

We wish to briefly describe the changes made to the original model. Costs associated with high-risk preterm patients refer to 24 months after enrollment instead of 14 years so as to uniform the financial evaluation to the new data available in literature [14]. Palivizumab administration scheme was changed, with three 50 mg vials and two 100 mg vials instead of the five 100 mg vials administered in the first model. Finally, all of the health care resources used by the patient were revaluated based on year 2007 costs and fees.

As regards general results, patients receiving palivizumab prophylaxis show better efficacy in terms both of LY (+ 0.088) and QALY (+ 0.159) compared to those who did not receive any prophylaxis. The best efficacy result achieved by palivizumab is confirmed also when differentiating patients per gestational age and presence of BPD.

On the other hand, in terms of the health care resources used, the mean cost per patient receiving prophylaxis (€ 6,244.20) was higher (+28.3%) compared to the estimated cost per patient without prophylaxis (€ 4,867.70). Therefore, due to the greater efficacy and greater costs associated with palivizumab versus the comparator (absence of prophylaxis), it was necessary to determine the incremental cost-effectiveness ratio per LY and QALY.

The calculation of the incremental cost per life year gained was based on an overall mean cost per patient of € 6,244.20 for palivizumab and of € 4,867.70 for absence of prophylaxis, with a mean survival of 29.842 years and 29.754 years, respectively. The incremental cost for prophylaxis (€ 1,376.50) was thus compared to an incremental efficacy of 0.088 years, leading to a cost per life year gained of € 15,568.65. In the case of the incremental cost per QALY the same mean total costs were recorded per patient (€ 6,244.20 and € 4,867.70), with however different outcomes. Palivizumab is characterized by 29.202 QALYs and absence of prophylaxis is associated with 29.043 QALYs. Incremental efficacy in this case is equal to 0.159 QALYs; therefore the incremental cost per QALY is € 8,676.74. Taking into account patient subgroups, the incremental cost effectiveness ratio per LY ranges between € 4,332.29 and € 28,417.08, whereas those per QALY range between € 2,731.81 and € 14,937.32.

All incremental costs calculated in this paper were finally compared against internationally acknowledged threshold values, which reflect the decision-making bodies' willingness to pay in order to achieve additional health units. Several international studies addressed the issue and established threshold values [22-25] and € 50,000 were set as the threshold below which a therapy is acceptable. Incremental costs per life year gained and per QALY calculated for palivizumab versus the absence of prophylaxis were always noted to be below the aforesaid threshold. The incremental cost per LY or per QALY of palivizumab was quite different observing the results of the three infant groups. The 33–35 weeks infant group showed the higher ICER per LY (€ 28,417.08) and per QALY (€ 14,937.32). If we considered a more restricted threshold value (< € 50,000), this third infant group (33–35 weeks) could be included in the prophylaxis programs, provided additional risk factors are present.

The univariate analyses conducted on several clinical and financial parameters taken into consideration in the model confirmed the robustness of the results, producing ICERs that were always below € 50,000. Also the threshold analysis conducted on the probability of an RSV hospitalization occurring for a patient receiving palivizumab prophylaxis estimated that to reach an incremental cost per LY or per QALY exceeding € 50,000 the efficacy of palivizumab would have to drop to non-realistic levels, *i.e*., down to efficacy levels equal to or even lower than those for patients without prophylaxis.

These results must be compared with those generated by other studies. Nuijten et al. [26] conducted in the United Kingdom an incremental cost effectiveness analysis for palivizumab in the prophylaxis of RSV infections in preterm infants with gestational age < 36 weeks and the possible presence of complications such as BPD or CHD (Congenital Heart Disease). The assessment was conducted from the perspective of the UK NHS (National Health Service), comparing administration of palivizumab to absence of prophylaxis. Applying a discount (3.5%) both to costs and outcome, an incremental cost per QALY equal to £ 16,720 for children with gestational age < 36 weeks and for children with BPD and an ICER per QALY of £ 6,664 for children with CHD. The results of the study conducted by Nuijten et al. [26] suggest that from the NHS viewpoint, palivizumab is cost effective compared to the therapeutic alternative represented by absence of prophylaxis.

Lazaro et al. [27] assessed efficacy and costs of palivizumab in the prevention of RSV infections in preterm children with gestational age in the 32–35 week range with two or more risk factors. The study was conducted from the perspective of the Spanish health care system, with absence of prophylaxis as the comparator. The Authors, thanks to a decision-making model, have calculated (with a 3% discount applied to efficacy and costs) an incremental cost effectiveness ratio per QALY of € 13,849. Once again, palivizumab was noted to be cost-effective versus absence of prophylaxis.

The study conducted in the USA by Elhassan et al. [28] intended to assess both the cost-effectiveness ratio of palivizumab as RSV prophylaxis in preterm children without BPD and the impact of decreasing recurrent wheezing risks in patients receiving prophylaxis on the cost effectiveness ratio. The Authors constructed two decision-making models, one of which took into account also risks of recurrent wheezing after an RSV infection and another one that did not consider this risk. The patients included in the model were preterm children with gestational age ranging from 26 to 32 weeks. Prophylaxis with palivizumab was compared against absence of prophylaxis. The results of the study conducted by Elhassan et al. [28] estimated that administration of palivizumab was cost effective only when taking into account also the benefits yielded by a decreased risk of recurrent wheezing in RSV infection patients.

The results of the present review present limitations and therefore several considerations must be taken into account when interpreting the data. As the study conducted by Simoes et al.[14] has estimated the probabilities of developing recurrent wheezing episodes during 24 months of follow-up after the enrollment in preterm children with gestational age equal or less than 35 weeks, with or without RSV hospitalization, the present model is based on the assumption that such probability was identical for all children, without differentiating based on gestational age and the presence of BPD. Moreover, since the study performed by Simoes et al.[14] did not report the odds of developing recurrent wheezing in preterm children receiving prophylaxis with RSV hospitalization, for this group of patients the same percentage (17%) calculated for preterm children with RSV hospitalization not receiving prophylaxis was conservatively adopted. To assess the impact of this double assumption, a sensitivity analysis was performed to test the probability of developing recurrent wheezing during the 24 months of follow-up post-enrollment, with a ± 10% change of the base value, assuming the worst case scenario for palivizumab; also in this case, the incremental cost effectiveness ratios per LY and per QALY show values lower than € 50.000.

Another limitation of the study might be that a pre-existing simulation model was adopted for the comparison between the two alternatives, and was adapted with clinical data deriving from international literature. This choice might be justified by the fact that to date no studies are available providing national data suitable for constructing the model population.

The dosage scheme (three 50 mg vials + two 100 mg vials) adopted herein for prophylaxis administration is different from that used in the original model.[15] In support thereof, a dosage of 15 mg of active principle per kg of weight was considered, assuming that average weight of the preterm infant in the first three months of life was less than 3.4 kg, thus such as to justify the administration of a 50 mg vial; moreover the model conservatively included also the non-administered drug. Moreover, by means of the sensitivity analysis, also the five 100 mg vials administration assumption was taken into consideration, and also in this case ICERs per LY and per QALY remained lower than the € 50,000 threshold.

Finally, as already pointed out by the original study, a further limitation might be the use of average costs referring to an adult sample for the appreciation of the annual mean cost for recurrent wheezing of pediatric patients. However, comparing the cost included in the present model (€ 1,226.88) against that of children suffering from asthma (US$ 1,129) within a study conducted in the United Stated in 1999 [29], we have reason to believe that the data we used are conservative.

## Conclusion

Based on the results of the present cost-effectiveness assessment it therefore appears possible to state that compared to absence of prophylaxis, the administration of palivizumab in preterm infants of varying gestational ages, with or without complications, does improve survival (quality-weighted or not) at reasonable costs in terms of resources covered by the NHS, when compared to internationally accepted threshold values. However, there is not general consensus concerning palivizumab prophylaxis for preterm infants born between 32 and 35 weeks of gestational age without chronic lung disease and haemodynamically significant congenital heart disease. [22].

## Abbreviations

BPD: Bronchopulmonary dysplasia; ICER: Incremental Cost Effectiveness Ratio; ICU: Intensive Care Unit; LY: Life-years; NHS: National Health System; OW: Ordinary ward; QALY: Quality-Adjusted Life-Years; RSV: Respiratory Syncytial Virus.

## Competing interests

The authors declare that they have no competing interests.

## Authors' contributions

All the authors (GC, RR and US) contributed in the conceiving, design and realization of the manuscript. All authors read and approved the final manuscript.
